# Mechanism of Non–Small Cell Lung Cancer–Derived Extracellular Vesicle miRNA hsa‐let‐7b‐5p Targeting AP1S1 to Regulate M2 Macrophage Polarization

**DOI:** 10.1155/ijog/8220478

**Published:** 2026-01-17

**Authors:** Lijuan Liu, Zixing Kou, Tianhua Wang, Qihang Shang, Qinxiang Zhang, Guanghui Liu, Jing Ai, Yanwen Zhao, Changgang Sun

**Affiliations:** ^1^ Department of Special Medicine, School of Basic Medicine, Qingdao University, Qingdao, China, qdu.edu.cn; ^2^ Department of Oncology, Weifang Traditional Chinese Hospital, Weifang, China, wfszyy.com; ^3^ Faculty of Chinese Medicine and State Key Laboratory of Quality Research in Chinese Medicine, Macau University of Science and Technology, Macao, China, must.edu.mo; ^4^ College of First Clinical Medicine, Shandong University of Traditional Chinese Medicine, Jinan, China, sdutcm.edu.cn; ^5^ College of Traditional Chinese Medicine, Shandong Second Medical University, Weifang, China

**Keywords:** AP1S1, M2 macrophage, miRNA hsa-let-7b-5p, non–small cell lung

## Abstract

**Background:**

Non–small cell lung cancer (NSCLC) accounts for over 80% of lung cancer cases. Further, the complex tumor immune microenvironment (TIME) is a critical factor in treatment resistance and poor prognosis associated with tumors. Tumor‐associated macrophages (TAMs), a major component of the TIME, significantly promote tumor progression through their polarization toward the immunosuppressive M2 phenotype. Reportedly, NSCLC cells regulate TAM polarization by secreting extracellular vesicles (EVs) to deliver miRNAs; however, the specific underlying molecular mechanisms remain unclear. In this study, we aimed to elucidate the regulatory role of miRNAs derived from NSCLC EVs in TAM polarization and explore potential novel therapeutic targets.

**Methods:**

Through high‐throughput sequencing and bioinformatics analysis, key regulatory targets were screened. Ki‐67 staining was employed to detect cell proliferation, flow cytometry was performed to analyze cell apoptosis, RT‐qPCR and Western blot were used to measure mRNA and protein expression levels, and Transwell assays were conducted to assess cell migration and invasion capabilities to investigate the molecular mechanisms underlying the miRNA‐mediated regulation of TAM polarization by NSCLC‐derived EVs.

**Results:**

NSCLC‐derived EVs were successfully isolated and characterized. Bioinformatics analysis of EVs′ miRNA sequencing data revealed that the hsa‐let‐7b‐5p/Adaptor‐Related Protein Complex 1 subunit sigma 1 (AP1S1) axis may be a key regulator of TAM polarization. In vitro experiments confirmed that the hsa‐let‐7b‐5p mimic potentially suppressed M2 polarization of TAMs via the AP1S1/p53 signaling axis, thereby attenuating the proliferation, migration, and invasion capabilities of NSCLC cells.

**Conclusion:**

This study revealed the molecular mechanism by which hsa‐let‐7b‐5p reshapes the immune microenvironment of NSCLC cells by targeting and inhibiting AP1S1 expression, thereby regulating the polarization of TAMs toward the M2 phenotype. Thus, the hsa‐let‐7b‐5p/AP1S1 axis may serve as a potential therapeutic target for NSCLC immunotherapy, providing novel strategies for improving patient prognosis.

## 1. Introduction

Lung cancer is the leading cause of cancer mortality worldwide [1–3]. Based on pathological characteristics, lung cancer is classified into small cell lung cancer (SCLC) and non–small cell lung cancer (NSCLC), with NSCLC accounting for over 80% of the lung cancer cases [4]. Although the widespread application of immunotherapy has significantly improved the overall survival (OS) of patients with NSCLC [5, 6], the 5‐year survival rate remains only 15.6% [7]. The current situation is primarily attributed to the highly complex tumor immune microenvironment (TIME) encountered in NSCLC [8].

TIME is a dynamic ecosystem comprising immune cells, stromal cells, extracellular matrix, and immunomodulatory factors [9–11]. Through an intricate network of interactions, it orchestrates pivotal biological processes, such as immune responses, tumor initiation and progression, and inflammatory reactions [12, 13]. Functionally, tumor‐associated macrophages (TAMs) can be categorized into the classically activated proinflammatory M1 phenotype and the alternatively activated anti‐inflammatory M2 phenotype. M1 macrophages exert antitumor effects and mediate pathogen clearance, whereas M2 macrophages are involved in tissue repair and immune suppression [14, 15]. Emerging evidence indicates that the dynamic interplay between the TIME and macrophage polarization modulates tumorigenesis and progression, rendering TAMs a promising therapeutic target [16]. Further investigations have revealed that transcription factors such as nuclear factor‐*κ*B (NF‐*κ*B), Krüppel‐like factors, and interferon regulatory factors determine macrophage polarization by regulating the expression of specific gene sets and that the activity of these transcription factors is finely tuned by microRNAs (miRNAs) [17]. miRNAs are ~22‐nucleotide noncoding RNA molecules that primarily modulate gene expression via mRNA degradation or translational repression [18].

Extracellular vesicles (EVs) are phospholipid bilayer nanostructures that are actively secreted by cells and are characterized by a typical cup‐shaped morphology. Their cargo comprises proteins, lipids, nucleic acids, and other bioactive molecules. Functioning as pivotal mediators of intercellular communication, EVs extensively modulate physiological and pathological processes within both local and systemic microenvironments [19–21]. Recent studies have demonstrated that NSCLC‐derived EVs can deliver specific miRNAs to orchestrate TAM polarization, thereby facilitating tumor proliferation, invasion, and chemoresistance [22–24]. However, the precise molecular mechanisms underlying EV‐mediated macrophage polarization remain unclear. In the present study, we isolated and characterized EVs secreted by the NSCLC cell line A549 and employed high‐throughput sequencing to profile differentially expressed miRNAs (DEmiRNAs) within these vesicles. Integrative bioinformatics analyses were subsequently performed to identify critical miRNA/mRNA regulatory axes. These axes may remodel the NSCLC immune microenvironment by influencing TAM polarization, thereby providing a theoretical foundation and potential therapeutic targets for the development of novel precision interventions.

## 2. Materials and Methods

### 2.1. Cell Culture

Human NSCLC lines A549 and H1299, human bronchial epithelial cell line BEAS‐2B, and human acute monocytic leukemia cell line THP‐1 were kindly provided by the Oncology Laboratory of Weifang Hospital of Traditional Chinese Medicine. All cells were maintained in RPMI‐1640 medium (Solarbio, China) supplemented with 10% fetal bovine serum (Fuxin Bio, China) and 1% penicillin–streptomycin (Solarbio, China) at 37°C in a humidified atmosphere containing 5% CO_2_.

### 2.2. Isolation and Characterization of EVs

After A549 cells had been cultured for 36–48 h, the conditioned medium was collected and subjected to sequential centrifugation at 4°C, 3000 × *g* for 10 min per cycle until no visible pellet remained, thereby eliminating cellular debris. Subsequently, EVs were harvested using a commercial EV isolation and purification kit (Umibio, China). Nanoparticle tracking analysis (NTA) was used to determine EV size distribution, transmission electron microscopy (TEM) was used to visualize EV morphology, and Western blotting was performed to verify the presence of canonical EV surface markers.

### 2.3. RNA Isolation, Library Preparation, and Sequencing

Small RNA libraries were constructed from the isolated EVs and subjected to paired‐end sequencing on an Illumina platform. EV‐derived miRNA profiles relevant to NSCLC were acquired, and expression levels were normalized using the transcripts per million algorithm.

### 2.4. Integrative Multiomics Data Analysis

#### 2.4.1. Data Acquisition and Preprocessing

Multidimensional analyses were performed by integrating The Cancer Genome Atlas Lung Adenocarcinoma (TCGA‐LUAD) cohort with the Gene Expression Omnibus (GEO) dataset GSE135918. The TCGA‐LUAD dataset encompasses RNA‐seq data (FPKM‐normalized) derived from 535 tumor tissues and 59 matched adjacent nontumor tissues. The GSE135918 dataset supplies miRNA expression profiles (platform GPL18058) obtained from five freshly resected lung cancer specimens and five corresponding nontumor lung tissues.

#### 2.4.2. Identification of DEmiRNAs and mRNAs

CIBERSORT, a deconvolution algorithm based on linear support vector regression, was employed to quantify the relative abundance of 22 human immune cell subpopulations from expression matrices, thereby delineating the immune cell disparities between LUAD patients and tumor‐free controls. Genes exhibiting significant correlations with M2 macrophage infiltration scores were retained (Pearson′s correlation |*r*| > 0.2, *p* < 0.01).

#### 2.4.3. Immune Infiltration Analysis

CIBERSORT, a deconvolution algorithm employing linear support vector regression, was applied to the expression matrices to quantify the relative proportions of 22 distinct human immune cell subtypes, thereby elucidating the immune cell discrepancies between LUAD patients and tumor‐free individuals [25]. Subsequently, only genes that exhibited a significant correlation with the M2 macrophage infiltration score were retained (Pearson′s correlation |*r*| > 0.2, *p* < 0.01).

#### 2.4.4. Protein Expression Profiling of AP1S1 and TNFRSF11A

Immunohistochemical images illustrating AP1S1 and TNFRSF11A expression in normal and LUAD tissues were retrieved from the Human Protein Atlas (HPA) database.

#### 2.4.5. Identification of Survival‐Associated Genes

M2 macrophage–associated genes in the TCGA cohort were first screened by univariate Cox regression analysis using R software, retaining those significantly correlated with OS (*p* < 0.05). Subsequently, the prognostic relevance of these candidate genes was independently validated using the online KM plotter platform. The KM plotter integrates publicly available datasets, including GEO, EGA, and TCGA, and provides Kaplan–Meier survival curves for visual and quantitative assessment of survival differences, thereby ensuring the accuracy and robustness of the selected gene signatures.

#### 2.4.6. Construction of the miRNA–mRNA Regulatory Network

Experimentally verified miRNA–target interactions were retrieved from TarBase (v9.0) and miRTarBase (v8.0), two extensively curated reference repositories that index only experimentally supported miRNA–mRNA pairs [26, 27]. The DEmiRNAs derived from our sequencing data and public datasets were intersected with predicted targets to establish high‐confidence regulatory axes. Molecular binding between candidate miRNAs and their cognate mRNAs was subsequently evaluated using RNAhybrid (v2.2) (https://bibiserv.cebitec.uni-bielefeld.de/rnahybrid), which calculates the minimum free energy of RNA duplex formation.

#### 2.4.7. Gene Set Enrichment Analysis (GSEA)

GSEA evaluates genome‐wide expression profiles by testing whether predefined gene sets are statistically enriched among differentially expressed genes. These sets comprised genes that shared common chromosomal locations, pathways, or functional annotations. The analyses were performed using the clusterProfiler R package (v3.5). Gene‐level fold‐change values were ranked to generate an ordered gene list, which was then analyzed against Gene Ontology (GO) and Kyoto Encyclopedia of Genes and Genomes (KEGG) gene sets. A false‐discovery‐rate‐adjusted *p* value < 0.05 was adopted as the significance threshold.

### 2.5. Cell Transfection

NSCLC cells were transfected with miR‐let‐7b‐5p mimic or mimic‐ctrl (NC; GenePharma, China) using Lipofectamine 2000 (Invitrogen, China).

### 2.6. Isolation and Cultivation of Macrophages

THP‐1 monocytes were differentiated into M0 macrophages by stimulation with 100 ng/mL phorbol 12‐myristate 13‐acetate (PMA; MEC) for 24 h. M0 macrophages were subsequently exposed for an additional 24 h to one of the following: (i) PBS, (ii) A549‐derived exosomes transfected with negative control mimic (A549‐Exo‐NC), (iii) A549‐derived exosomes transfected with miR‐let‐7b‐5p mimic (A549‐Exo‐miR‐let‐7b‐5p), or (iv) 20 ng/mL IL‐4/IL‐13. After treatment, the surface expression of the macrophage markers CD206 (M2 marker) and CD86 (M1 marker) was quantified by flow cytometry (Agilent, China) using fluorochrome‐conjugated monoclonal antibodies (BD Biosciences, United States).

### 2.7. Reverse Transcription‐Quantitative Polymerase Chain Reaction (RT‐qPCR)

Total RNA was isolated from cell lines or tissues using TRIzol R reagent (CWBio, China). The PrimeScript RT reagent kit (AKR bioscience, China) was used to reverse‐transcribe total RNA into cDNA. RT‐qPCR was performed on a fluorescence quantitative PCR instrument (Bio‐Rad, China) using the SYBR Green Premix Pro Taq HS qPCR Kit, according to the following protocol: 5 min at 95°C, followed by 40 cycles (10 s at 95°C and 30 s at 60°C). Primer sequences were as follows: miRNA‐specific stem‐loop primer, 5 ^′^‐GTCGTATCCA‐GTGCAGGGTCCGAGGTATTCGCACTGG ‐ATACGACTCACACT‐3 ^′^ for reverse transcription; stem‐loop primer, 5 ^′^‐GTCGTATC ‐CAGTGCAGGGTCCGAGGTATTC‐GCACTGGATACGACTCACACT‐3 ^′^; miR‐let‐7b‐5p forward primer sequence, CGGAAGAAACAACAACCACG; reverse primer sequence, AGTGCAGGGTCCG‐AGGTATT; and reverse primer, GTCGTATCCAGTGCAGGGTCCGAGGTATTCGCA‐CTGGATACGACCGGGGG for qPCR.

### 2.8. CCK‐8 Assay

A 100 *μ*L suspension of pretreated A549 cells (1 × 10^4^ cells per well) was seeded into each well and incubated overnight at 37.0°C. Subsequently, 10 *μ*L of CCK‐8 solution was added to each well and incubated for 4 h at 37.0°C. Absorbance was measured at 450 nm using a microplate reader (Thermo Fisher Scientific, China).

### 2.9. Cell Apoptosis Detection

Non–small cell lung carcinoma cells were centrifuged at 500 g for 5 min (1 × 10^6^ cells/mL), and the resulting pellet was gently resuspended. Cells were then incubated with 5 *μ*L of Annexin V–PE and 7‐AAD staining solution for 15 min at room temperature in the dark. Apoptosis was assessed using a flow cytometer (Agilent, China) and quantified using the FlowJo software (Agilent).

### 2.10. Western Blotting

Total cellular protein was extracted using RIPA lysis buffer (CWBio, China). Proteins (40 *μ*g per lane) were separated on 10% SDS‐PAGE gels and transferred onto PVDF membranes. After blocking with 5% nonfat milk for 30 min, membranes were incubated overnight at 4°C with the following primary antibodies: anti‐CD63 (1:1000; Abcam), anti‐TSG101 (1:1000; Abcam), anti‐CD9 (1:1000; Abcam), E‐cadherin (1:50,000; PTG), N‐cadherin (1:5000; PTG), vimentin (1:50,000; PTG), GAPDH (1:10,000; PTG), AP1S1 (1:4000; PTG), MDM2 (1:2000; PTG), p53 (1:3000; PTG), and c‐MYC (1:10,000; PTG). Membranes were then incubated with horseradish peroxidase–conjugated goat antirabbit (1:5000; Abcam) or goat antimouse (1:5000, Abcam) secondary antibodies for 1 h at room temperature. Protein bands were visualized using an ECL detection kit (Meilun Bio).

### 2.11. Immunofluorescence

Non–small cell lung carcinoma cells were fixed in methanol for 20 min and incubated with primary antibodies against Ki‐67 (1:1000; Abcam) or PKH67 (1:1000; Abcam). After washing, cells were incubated with the corresponding secondary antibodies (goat antirabbit IgG; 1:5000, Abcam) and examined under a fluorescence microscope (Leica, China).

### 2.12. Dual‐Luciferase Reporter Assay

On the day of transfection, two solutions were prepared: Solution A contained 50 ng reporter plasmid, 150 ng miRNA expression plasmid, and 10 ng Opti‐MEM (Yuchun, China), and Solution B contained 10 *μ*g Opti‐MEM and 0.2 *μ*L Lipofectamine 2000 (Invitrogen, China). After incubating for 5 min at room temperature, Solutions A and B were combined and incubated at room temperature for 20 min. A 20 *μ*L aliquot of the transfection complex was added to each well, and cells were cultured at 37°C in 5% CO_2_. Luciferase activity was measured using a Dual‐Luciferase Reporter Assay System (Promega, China).

### 2.13. Transwell Assay

For invasion assays, the upper chamber was precoated with 100 *μ*L Matrigel (BD BioCoat); migration assays were performed without Matrigel. A 200 *μ*L cell suspension was seeded into the upper chamber, and the lower chamber was filled with medium containing 10% FBS. After 24 h of incubation, cells on the lower surface were fixed, stained with 0.1% crystal violet, observed, and counted under an optical microscope.

### 2.14. Enzyme‐Linked Immunosorbent Assay (ELISA)

The concentrations of IL‐10 and TGF‐*β* in the supernatants of NSCLC cells were quantified using commercial ELISA kits (Fanke, China) according to the manufacturer′s protocols.

### 2.15. Statistical Analysis

All in vitro experiments were performed in triplicate. Data are expressed as mean ± SEM for parametric variables or as medians with ranges for nonparametric variables. Statistical analyses were conducted using SPSS Version 26.0 (IBM Corp., United States), and a two‐tailed *p* value < 0.05 was considered statistically significant.

## 3. Results

### 3.1. EV Isolation, Characterization, and miRNA Sequencing

EVs were harvested from the supernatant of the LUAD cell line A549 and the human lung epithelial cell line BEAS‐2B. TEM revealed the presence of characteristic cup‐shaped particles with diameters ranging from 30 to 150 nm (Figure 1a). NTA confirmed a similar exosome size distribution profile (Figure 1b). Furthermore, Western blot analysis validated three positive exosomal biomarkers, TSG101, CD63, and CD9, all of which were detected at their expected molecular weights in the corresponding positive control bands (Figure 1c) [28].

Figure 1Extracellular vesicle (EV) isolation, characterization, and miRNA sequencing. (a) Representative TEM image of EVs (scale bar = 100 nm). (b) NTA analysis of EV nanoparticle size distribution and particle concentration. (c) Western blot detection of canonical exosomal biomarkers (TSG101, CD63, and CD9) in EVs. CD9/TSG101 used HeLa cells as positive controls, while CD63 employed 293T cells as positive controls, all previously validated for positive protein expression. (d, e) Heatmap and volcano plot of differentially expressed miRNAs between A549 and BEAS‐2B cells.

**Figure:**
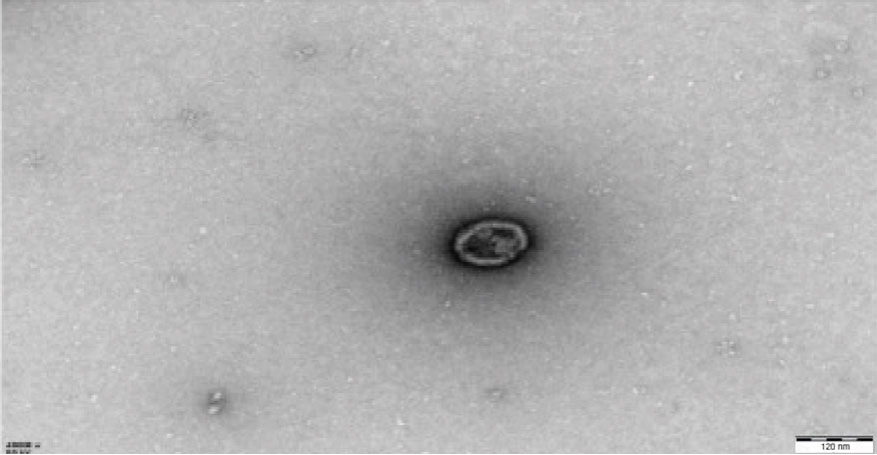
(a)

**Figure:**
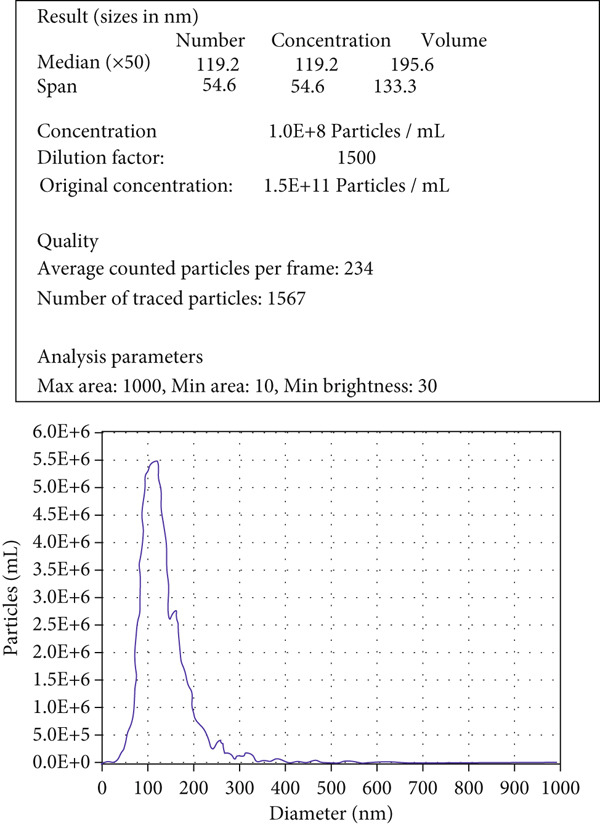
(b)

**Figure:**
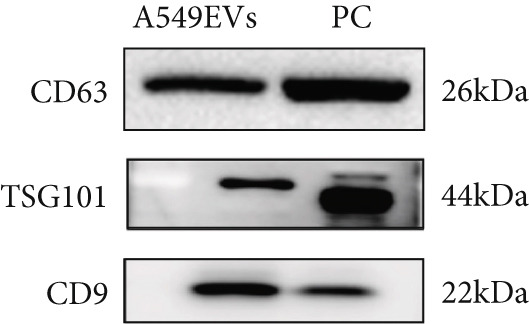
(c)

**Figure:**
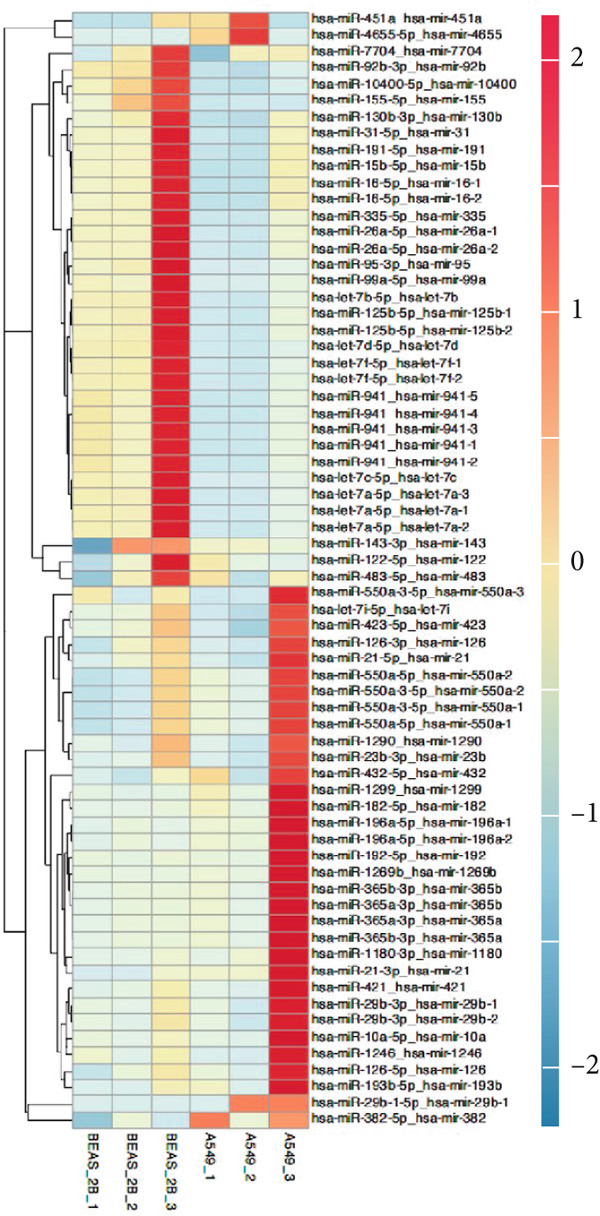
(d)

**Figure:**
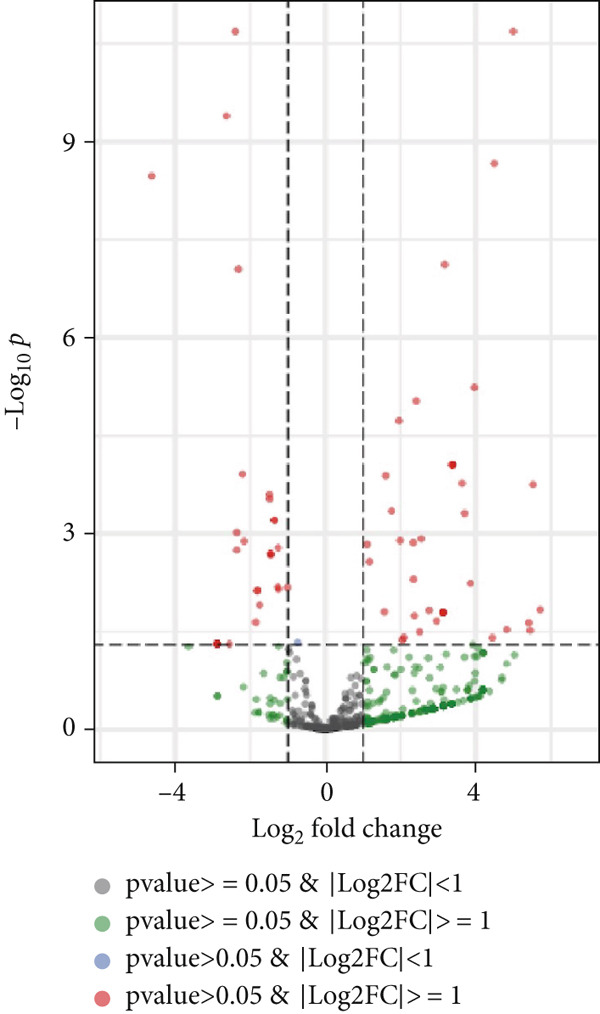
(e)

Subsequently, miRNA sequencing was performed on the EVs isolated from A549 and BEAS‐2B cells to identify potential regulatory targets (Figure 1d). A total of 67 DEmiRNAs (*p* value < 0.05 and |Log_2_FC| > = 1) were identified. Specifically, 38 miRNAs (e.g., hsa‐miR‐4655‐5p) were significantly upregulated in A549‐derived EVs, whereas 29 miRNAs (e.g., hsa‐miR‐let‐7b‐5p) were significantly downregulated.

### 3.2. Screening of DEmiRNAs and mRNA

We first performed a differential expression analysis based on the TCGA‐LUAD transcriptomic dataset. Using adjacent nontumor tissues as controls, 5404 differentially expressed genes were identified; of these, 3399 were significantly upregulated, and 2005 were significantly downregulated (Figure 2a).

Figure 2Screening of differentially expressed mRNAs in lung adenocarcinoma (LUAD). (a) Volcano plot visualizing differentially expressed genes (DEGs) in LUAD patients, with AP1S1 and TNFRSF11A highlighted as key candidates. (b) Quantitative comparison of AP1S1 mRNA expression levels between normal lung tissues and LUAD tumor tissues. (c) Quantitative comparison of TNFRSF11A mRNA expression levels between normal lung tissues and LUAD tumor tissues. (d) Immune cell infiltration analysis of AP1S1 expression in LUAD patients versus that in healthy cohorts, assessed via computational deconvolution (e.g., CIBERSORT). (e) Immune cell infiltration analysis of TNFRSF11A expression in LUAD patients versus that in healthy cohorts, assessed via computational deconvolution. (f) Representative immunohistochemical (IHC) staining images showing AP1S1 and TNFRSF11A protein localization and intensity in normal lung tissues and LUAD tumor tissues.

**Figure:**
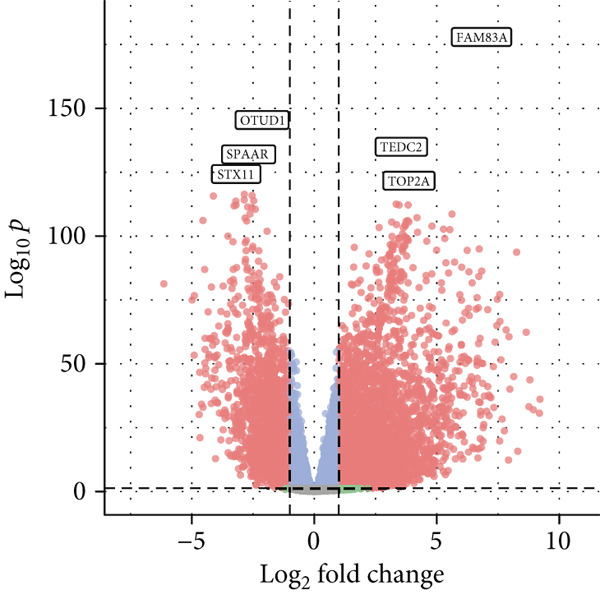
(a)

**Figure:**
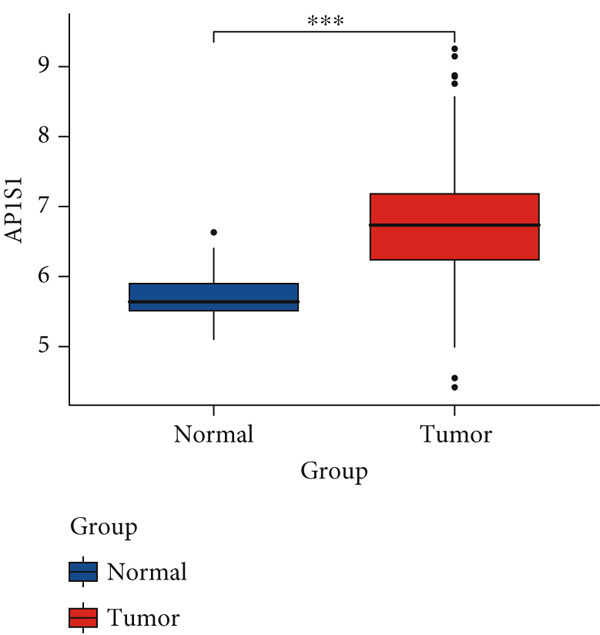
(b)

**Figure:**
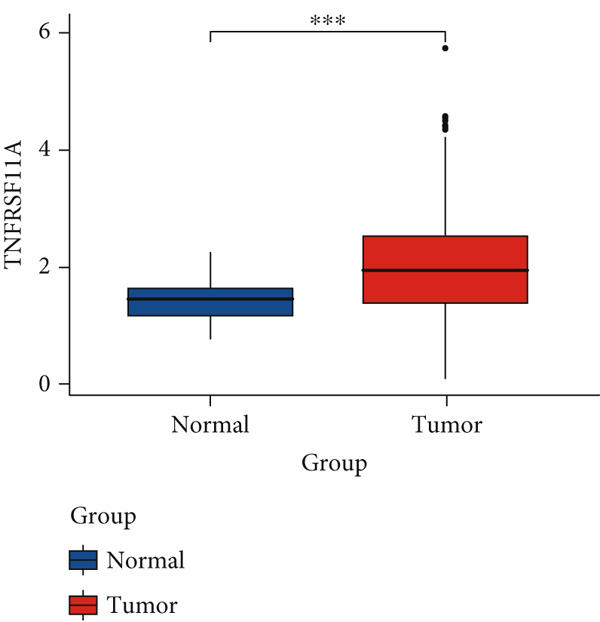
(c)

**Figure:**
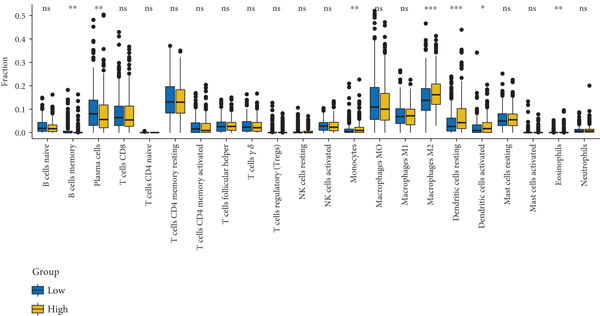
(d)

**Figure:**
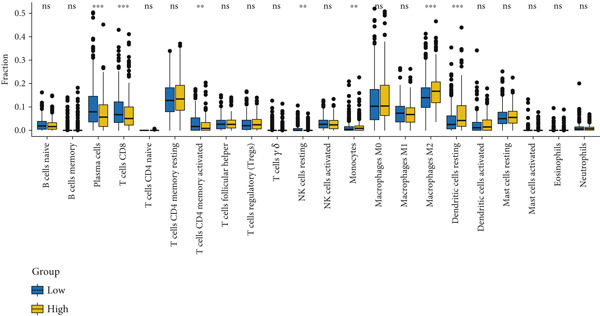
(e)

**Figure:**
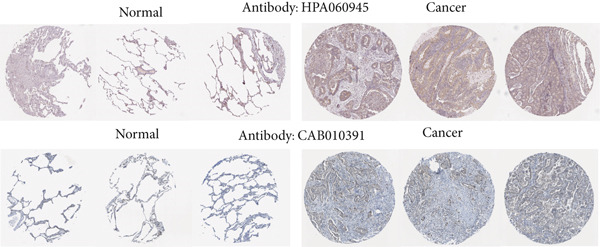
(f)

Further analyses were performed using the CIBERSORT algorithm to assess the correlation between differential gene expression and M2 macrophage infiltration. We identified 499 M2 infiltration–associated genes (screening criterion: absolute value of Spearman′s correlation coefficient > 0.2).

Univariate Cox proportional hazards regression analysis revealed that 121 genes were significantly associated with patient OS (*p* < 0.05). Among these, AP1S1 and TNFRSF11A exhibited significantly higher expression in lung cancer tissues than in normal tissues (Figure 2b,c). Moreover, their high expression was significantly and positively correlated with both increased M2 macrophage infiltration (Figure 2d) and poor patient prognosis (Figure 2e).

Immunohistochemical validation using the HPA confirmed markedly elevated protein expression levels of AP1S1 and TNFRSF11A in LUAD tissues (Figure 2f).

### 3.3. Screening of miRNA–mRNA Regulatory Pairs

Differential expression analysis was performed using the GSE135918 dataset (a LUAD miRNA expression profile dataset from the GEO database) with normal tissues used as controls. We identified 239 DEmiRNAs; of these, 136 were significantly downregulated, and 103 were significantly upregulated (Figure 3a).

Figure 3Screening of miRNA–mRNA regulatory pairs in lung adenocarcinoma (LUAD). (a) Volcano plot depicting differentially expressed miRNAs (DEMs) in LUAD patients and healthy cohorts, with threshold lines indicating statistical significance (*p* < 0.05) and fold change ($\text{Lo}{\text{g}}_{2}\text{FC}\geq 1$ or ≤−1). (b–e) Venn diagrams illustrating the overlap between (b) upregulated DEMs and predicted target miRNAs of AP1S1, (c) upregulated DEMs and predicted target miRNAs of TNFRSF11A, (d) downregulated DEMs and predicted target miRNAs of AP1S1, and (e) downregulated DEMs and predicted target miRNAs of TNFRSF11A. (f) Survival analysis curves (Kaplan–Meier plots) showing the prognostic correlation between target miRNA/mRNA expression levels and overall survival in LUAD patients, with hazard ratios (HRs) and *p* values indicated. (g) GSEA pathway enrichment analysis demonstrating significant enrichment of AP1S1‐related genes in the p53 upregulation signaling pathway (normalized enrichment score [NES] and false discovery rate [FDR] *q*‐value provided).

**Figure:**
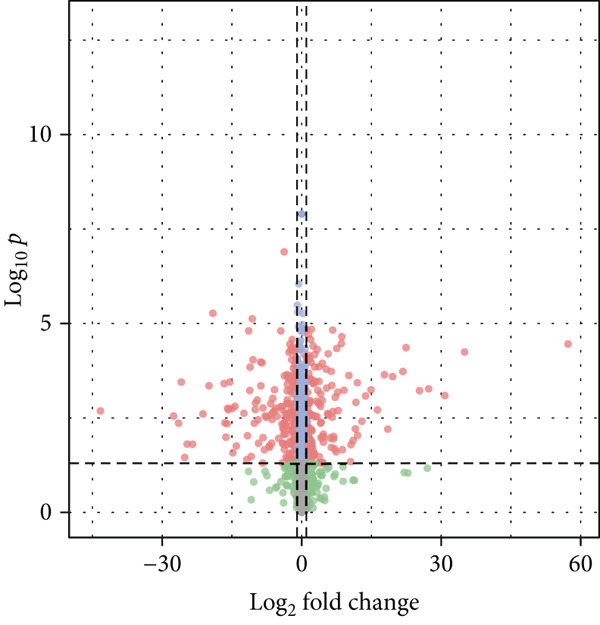
(a)

**Figure:**
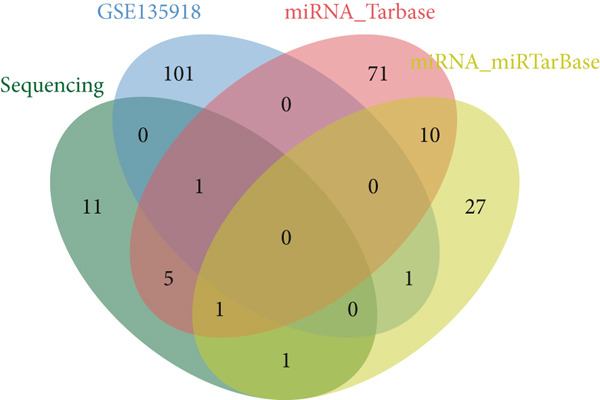
(b)

**Figure:**
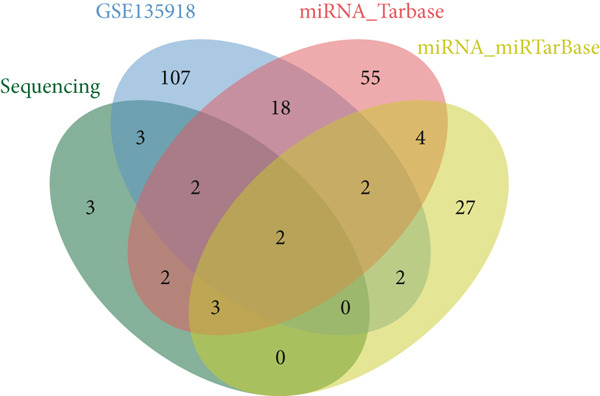
(c)

**Figure:**
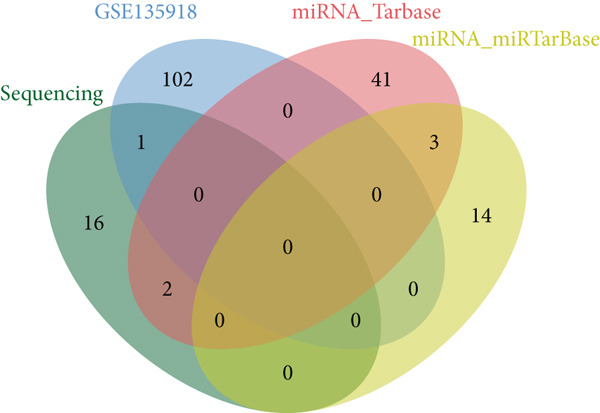
(d)

**Figure:**
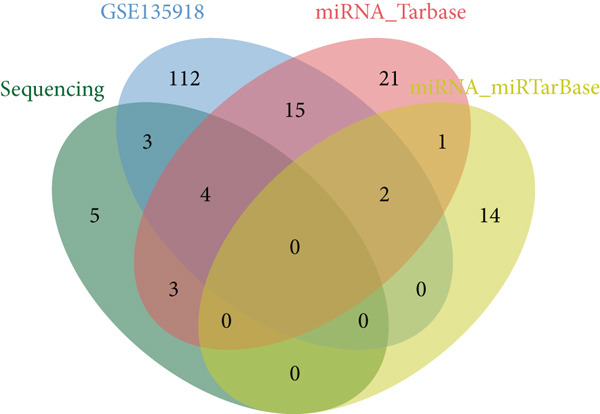
(e)

**Figure:**
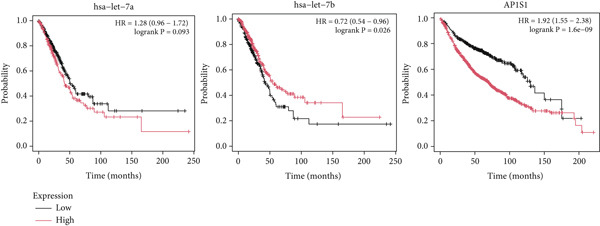
(f)

**Figure:**
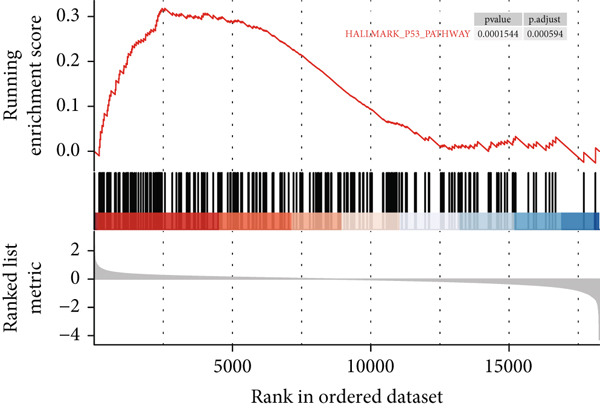
(g)

Cross‐validation using our group′s previous exosomal miRNA sequencing data (A549/BEAS‐2B) revealed eight overlapping DEmiRNAs. Potential upstream regulatory miRNAs for AP1S1 and TNFRSF11A were predicted using the TargetScan and miRDB databases, respectively. Intersecting these predictions with the DEmiRNAs identified two miRNA–mRNA regulatory pairs: hsa‐let‐7a‐5p/AP1S1 and hsa‐let‐7b‐5p/AP1S1 (Figures 3b, 3c, 3d, and 3e).

Survival analysis demonstrated that low expression of hsa‐let‐7b‐5p (hazard ratio [HR] = 0.72, *p* < 0.05) and high expression of AP1S1 (HR = 1.92, *p* < 0.05) were significantly associated with shortened OS in LUAD patients (Figure 3f). Functional modeling suggested that the hsa‐let‐7b‐5p/AP1S1 regulatory axis may improve patient prognosis by inhibiting M2 macrophage infiltration (correlation coefficient *r* = −0.20, *p* < 0.05).

We propose that modulation of the hsa‐let‐7b‐5p/AP1S1 axis may reduce M2 cell infiltration, ultimately leading to improved survival outcomes. Subsequent NAhybrid analysis revealed a high‐affinity binding site for hsa‐let‐7b‐5p within the 3 ^′^ untranslated region (3 ^′^UTR) of the target gene AP1S1, with a minimal free energy (mfe) of −29.3 kcal/mol, indicating a high‐confidence regulatory interaction. GSEA was employed to explore the pathways regulated by AP1S1, which showed significant enrichment in the p53 signaling pathway (Figure 3g).

### 3.4. miR‐let‐7b‐5p Suppresses Proliferation, Invasion, and Migration of NSCLC Cells

To investigate the effect of miR‐let‐7b‐5p on the biological behavior of NSCLC cells, we transfected miR‐let‐7b‐5p mimics into A549 and H1299 cells. Compared with the blank control and negative control (NC) groups, NSCLC cells in the miR‐let‐7b‐5p mimic group exhibited significantly suppressed proliferation, showing a time‐dependent inhibitory effect (Figure 4a). Assessment of proliferative activity via Ki‐67 staining confirmed that the proliferation rate of NSCLC cells in the miR‐let‐7b‐5p mimic group was markedly lower than that in the NC group (Figure 4b). Flow cytometric analysis of apoptosis revealed that at 72 h posttransfection, the apoptosis rate of NSCLC cells in the miR‐let‐7b‐5p mimic group was significantly higher than that in both the blank control and NC groups (Figure 4c). Transwell migration assays demonstrated that the overexpression of miR‐let‐7b‐5p significantly attenuated the migratory capacity of NSCLC cells. Furthermore, cell invasion was potently inhibited in the presence of miR‐let‐7b‐5p overexpression (Figure 4d). Collectively, these findings indicated that the miR‐let‐7b‐5p mimic suppressed the proliferation, migration, and invasion capabilities of NSCLC cells.

Figure 4miR‐let‐7b‐5p induces apoptosis and inhibits proliferation, migration, and invasion in NSCLC cells. (a–c) Cell proliferation and apoptosis in the blank control group, the NC group, and the miR‐let‐7b‐5p mimic group were assessed using CCK‐8 assay, Ki‐67 immunofluorescence, and flow cytometry, respectively. (d) The migration and invasion abilities of NSCLC cells in the NC group and the miR‐let‐7b‐5p mimic group were evaluated by Transwell chamber assay. (*N* = 3;  ^∗^ *p* < 0.05,  ^∗∗^ *p* < 0.01, and  ^∗∗∗^ *p* < 0.001).

**Figure:**
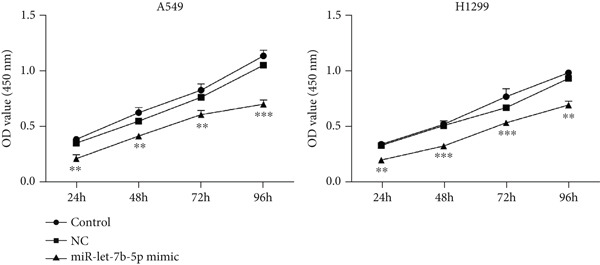
(a)

**Figure:**
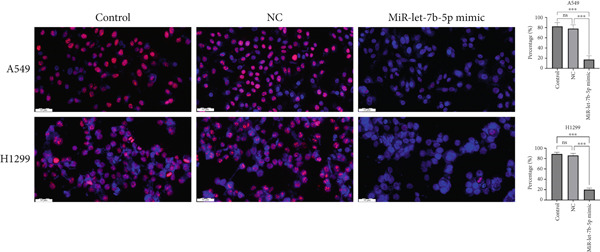
(b)

**Figure:**
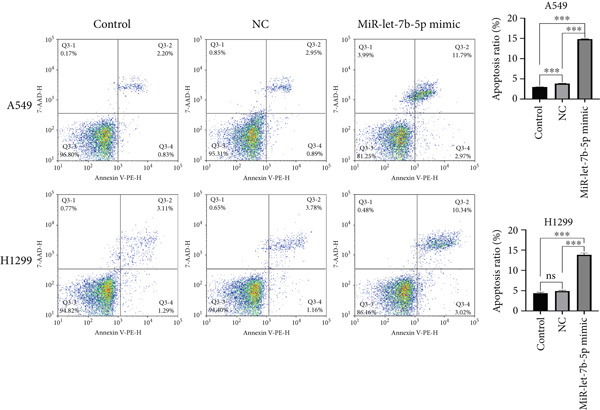
(c)

**Figure:**
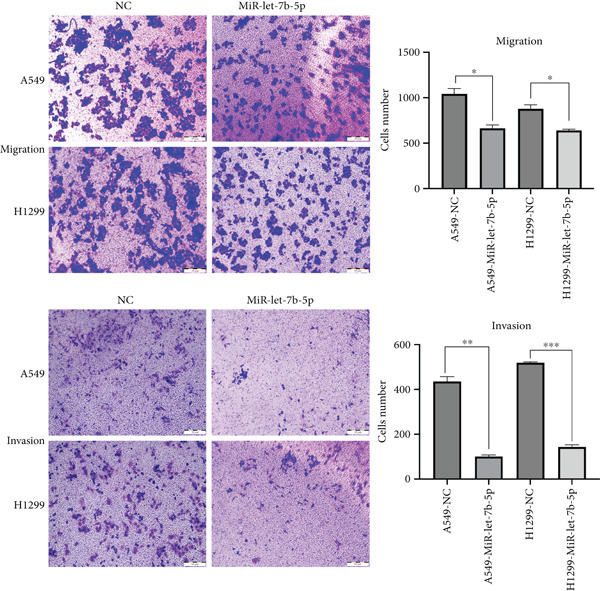
(d)

### 3.5. Lung Cancer–Derived Exosomal miR‐let‐7b‐5p Inhibits M2 Macrophage Polarization

To investigate whether exosomes mediated intercellular crosstalk by transferring miR‐let‐7b‐5p from A549 cells to macrophages (PMA‐differentiated human THP‐1 monocytes), we cocultured macrophages with PKH67‐labeled A549‐derived exosomes for 48 h. The PKH67 lipid dye was observed in macrophages, confirming that A549‐Exo cells could be transferred to macrophages (Figure 5a,b). To validate specific miRNA transport, exosomes derived from A549 cells transfected with a Cy5‐labeled miR‐let‐7b‐5p mimic were used in the coculture. Dual‐channel fluorescence imaging successfully captured the colocalization of the PKH67 membrane dye and Cy5‐miRNA within macrophages. In contrast, while PKH67 lipid staining was observed in the exosome‐negative control (Exo‐NC) group, no Cy5 fluorescence was detected, confirming that exosomes could deliver functional miR‐let‐7b‐5p intact to target cells (Figure 5a,b). Mechanistic validation experiments revealed that compared with normal A549 conditioned medium, the miR‐let‐7b‐5p levels showed no significant decrease after treatment with RNase A alone. However, combined treatment with RNase A and Triton X‐100 resulted in a significant reduction in the miR‐let‐7b‐5p content (Figure 5c), demonstrating the protective role of the exosomal membrane structure for its cargo. Functional assays further demonstrated that the miR‐let‐7b‐5p mimic significantly elevated the intracellular levels of internalized miR‐let‐7b‐5p in macrophages.

Figure 5Exosomal miR‐let‐7b‐5p from A549 cells is internalized by macrophages and inhibits their M2 polarization. (a, b) Immunofluorescence visualization of exosome–macrophage colocalization. (c) RT‐qPCR analysis of miR‐let‐7b‐5p levels in macrophages or miR‐let‐7b‐5p conditioned medium (CM) treated with RNase alone or RNase + Triton X‐100. (d) Flow cytometry evaluation of macrophage polarization status following treatment with various exosomes. (*N* = 3;  ^∗^ *p* < 0.05,  ^∗∗^ *p* < 0.01, and  ^∗∗∗^ *p* < 0.001).

**Figure:**
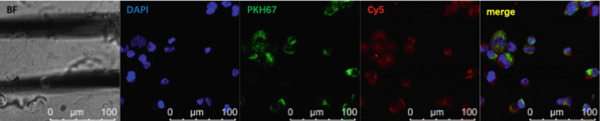
(a)

**Figure:**
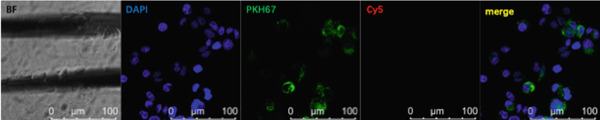
(b)

**Figure:**
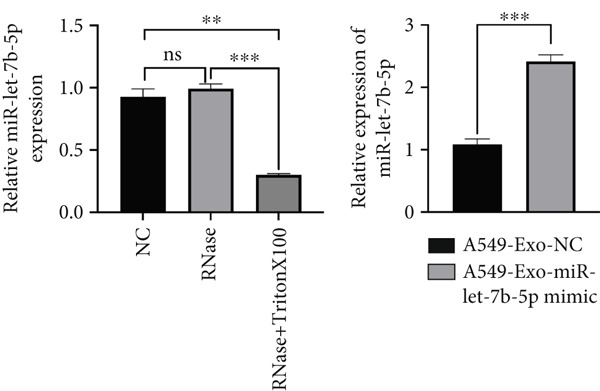
(c)

**Figure:**
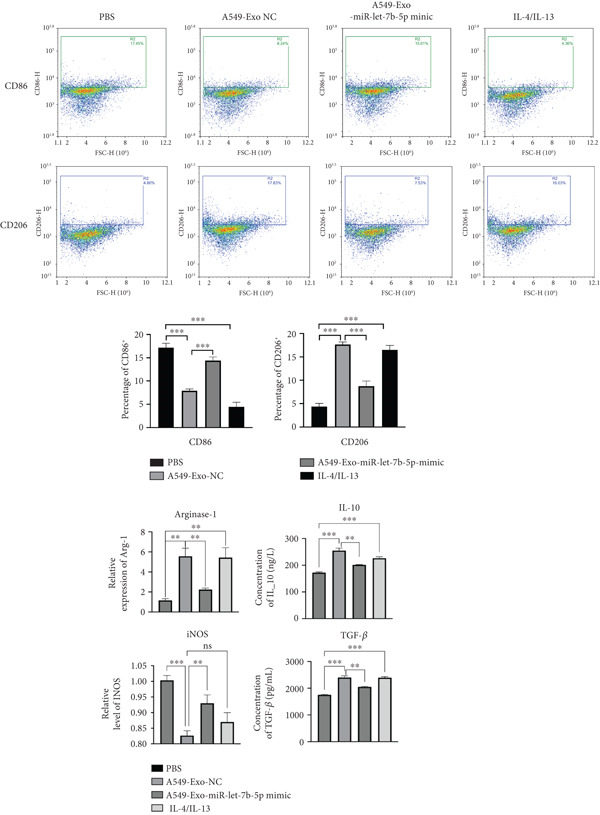
(d)

To investigate the role of miR‐let‐7b‐5p in the polarization of M2 phenotype macrophages, macrophages were treated with 30 *μ*g/mL of A549 cell–derived exosomes. The results showed that compared with the Exo‐NC group, macrophages in the Exo‐miR‐let‐7b‐5p mimic treatment group exhibited significant upregulation of M1 polarization markers (CD86, iNOS) and downregulation of M2 polarization markers (CD206, Arginase‐1, TGF‐*β*, and IL‐10) (Figure 5d). These results indicate that exosomal miR‐let‐7b‐5p effectively inhibits M2 macrophage polarization in lung cancer.

### 3.6. Lung Cancer–Derived Exosomal miR‐let‐7b‐5p Significantly Suppresses A549 Cell Migration, Invasion, and Epithelial–Mesenchymal Transition (EMT) by Inhibiting M2 Macrophage Polarization

To investigate whether lung cancer exosome–derived miR‐let‐7b‐5p inhibits the migration and invasion abilities of A549 cells by suppressing M2 macrophage polarization, we cocultured differently treated macrophages with A549 cells. Transwell assays demonstrated that compared with the A549+M^Exo-NC^ group, the A549+M^Exo-miR-let-7b-5p^ mimic group exhibited significantly reduced levels of cell migration and significantly reduced levels of cell invasion (Figure 6a,b). Furthermore, Western blot analysis revealed that relative to the A549+M^Exo-NC^ group, the A549+M^Exo-miR-let-7b-5p^ mimic group displayed significantly decreased protein levels of N‐cadherin and vimentin and significantly increased protein levels of E‐cadherin (Figure 6c). These findings suggest that exosomal miR‐let‐7b‐5p significantly inhibits A549 cell migration, invasion, and EMT process by suppressing M2 macrophage polarization.

Figure 6miR‐let‐7b‐5p suppresses A549 cell migration and invasion by inhibiting M2 macrophage polarization. (a, b) The migration and invasion abilities of A549 cells treated with different macrophage‐conditioned media were evaluated by the Transwell assay. (c) Meanwhile, the protein expression levels of E‐cadherin, N‐cadherin, and vimentin in A549 cells treated with different macrophage‐conditioned media were detected by Western blot. (*N* = 3;  ^∗^ *p* < 0.05,  ^∗∗^ *p* < 0.01, and  ^∗∗∗^ *p* < 0.001).

**Figure:**
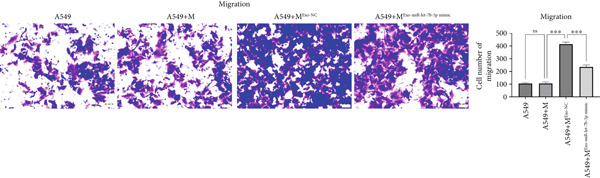
(a)

**Figure:**
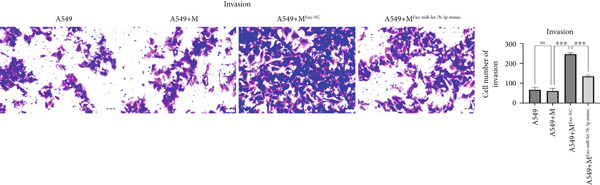
(b)

**Figure:**
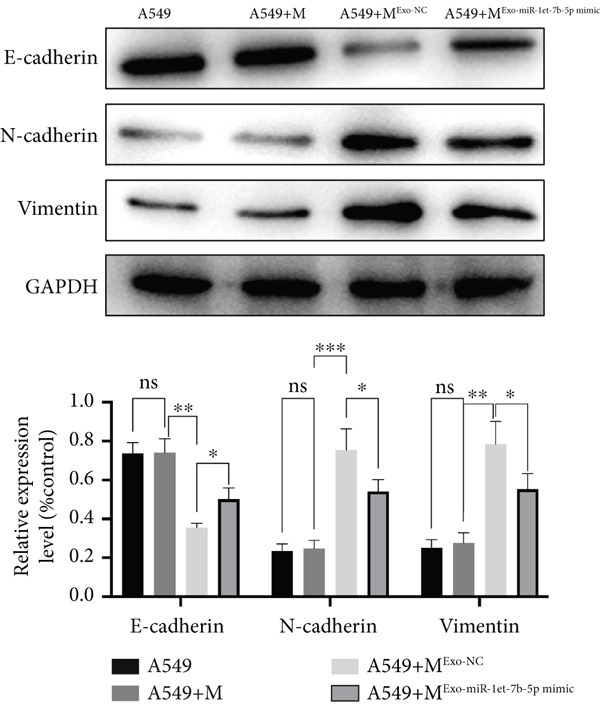
(c)

### 3.7. Lung Cancer–Derived Exosomal miR‐let‐7b‐5p Suppresses AP1S1 Expression

Previously, we identified AP1S1 as a potential target of miR‐let‐7b‐5p through high‐throughput sequencing and bioinformatics analyses. To validate this interaction, we performed dual‐luciferase reporter assays demonstrating that lung cancer–derived exosomal miR‐let‐7b‐5p regulates AP1S1 expression through specific binding to its 3 ^′^UTR region (nucleotide sequence: AGGCUGGA). In the wild‐type AP1S1 3 ^′^UTR (WT‐AP1S1) reporter system, miR‐let‐7b‐5p mimic significantly inhibited luciferase activity (Figure 7a). This repression was completely abolished when the binding site was mutated (MT‐AP1S1), confirming a direct regulatory interaction. Consistently, Western blot revealed that treatment with the miR‐let‐7b‐5p mimic significantly reduced protein levels of AP1S1 in macrophages (Figure 7b). These results demonstrated that A549 cell–secreted exosomal miR‐let‐7b‐5p effectively suppressed AP1S1 expression in macrophages.

Figure 7AP1S1 is a direct target of lung cancer–derived exosomal miR‐let‐7b‐5p. (a) Relative luciferase activity was detected by dual‐luciferase reporter assay. (b, c) The protein expression levels of AP1S1 and its downstream proteins (c‐MYC, MDM2, and p53) in the macrophages cultured under different conditions were detected by Western blot. (d, e) Cell viability and apoptosis of A549 cells treated with these different macrophage‐conditioned media were assessed using the CCK‐8 assay and flow cytometry, respectively. (f, g) Meanwhile, the invasion and migration abilities of these A549 cells were evaluated by the Transwell assay. (*N* = 3;  ^∗^ *p* < 0.05,  ^∗∗^ *p* < 0.01, and  ^∗∗∗^ *p* < 0.001).

**Figure:**
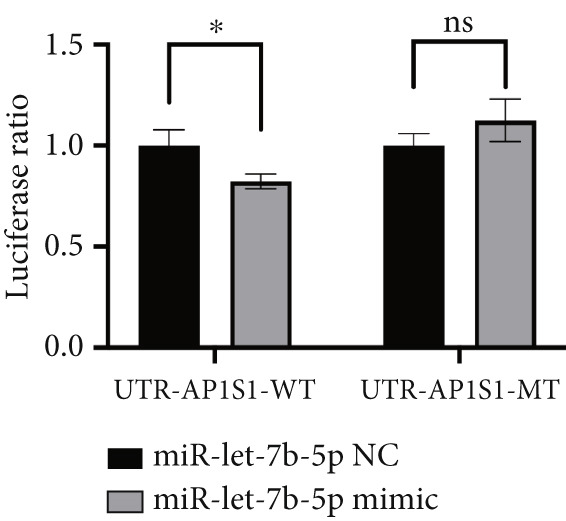
(a)

**Figure:**
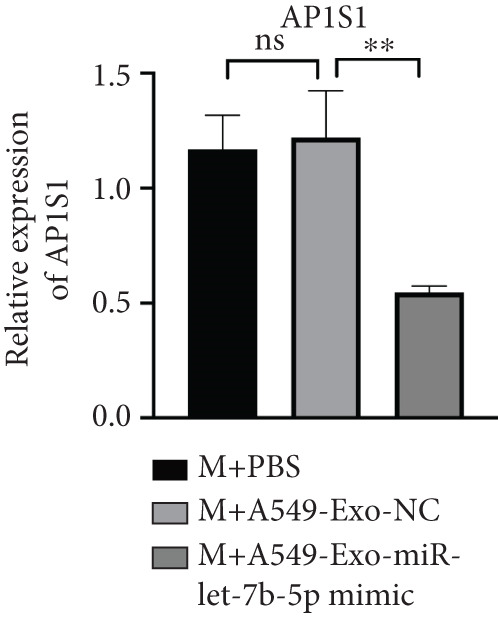
(b)

**Figure:**
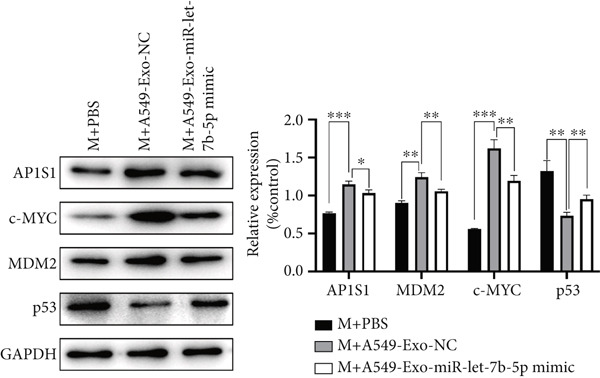
(c)

**Figure:**
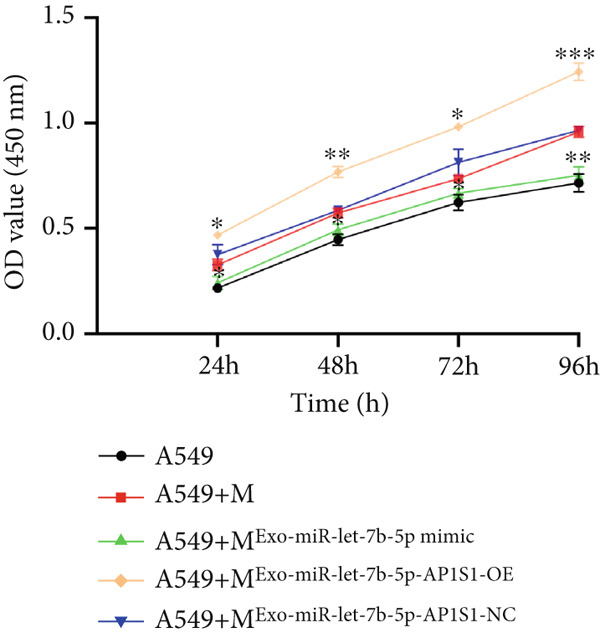
(d)

**Figure:**
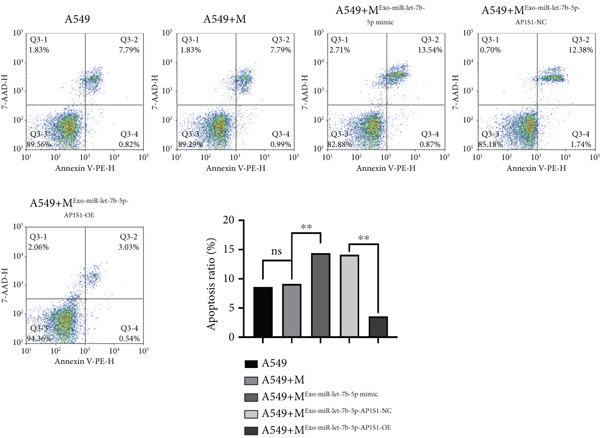
(e)

**Figure:**
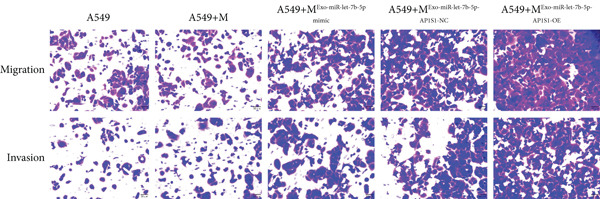
(f)

**Figure:**
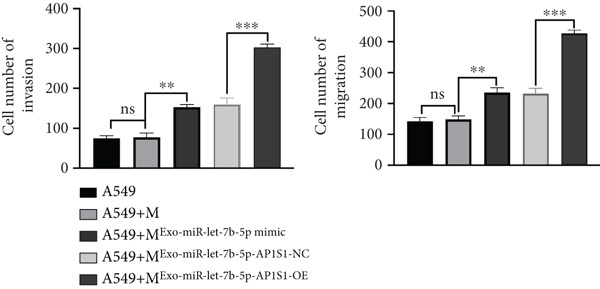
(g)

As the primary negative regulator of P53, MDM2 promotes its degradation through ubiquitination, forming a negative feedback loop [29]. To further validate whether Exo‐miR‐let‐7b‐5p regulates macrophage polarization via the AP1S1‐MDM2/c‐MYC‐TP53 axis, we examined the expression of relevant pathway proteins in macrophages. The results showed that compared with the M+A549‐Exo‐NC group, the M+A549‐Exo‐miR‐let‐7b‐5p mimic group exhibited significantly reduced protein levels of MDM2 and c‐Myc, whereas the protein levels of p53 were markedly increased (Figure 7c). Collectively, these findings suggest that the AP1S1/p53 axis may serve as a critical pathway through which miR‐let‐7b‐5p regulates macrophage function.

### 3.8. Inhibitory Effects of Lung Cancer–Derived Exosomal miR‐let‐7b‐5p on Tumor Cell Proliferation and Metastasis

To further demonstrate the interaction between AP1S1 and miR‐let‐7b‐5p, we overexpressed AP1S1 in macrophages and found that upon cotreatment with the A549‐Exo‐miR‐let‐7b‐5p mimic, AP1S1‐OE significantly counteracted the tumor‐suppressive effects of lung cancer exosomal miR‐let‐7b‐5p (Figure 7d). Moreover, compared with the macrophages treated solely with the A549‐Exo‐miR‐let‐7b‐5p mimic, those treated with both the A549‐Exo‐miR‐let‐7b‐5p mimic and AP1S1 overexpression markedly reversed the proapoptotic effects of miR‐let‐7b‐5p on A549 cells (Figure 7e) and enhanced the migration and invasion capabilities of A549 cells (Figure 7f,g). Collectively, these findings demonstrated that exosomal miR‐let‐7b‐5p derived from A549 cells inhibits AP1S1 expression, thereby modulating M2 polarization of TAMs to suppress tumor invasion and metastasis.

## 4. Discussion

In recent years, EVs have garnered significant attention because of their substantial potential for tumor diagnosis and therapy. Numerous studies have demonstrated that EVs carry various bioactive molecules, including DNA, RNA, proteins, and lipids, and play pivotal regulatory roles in tumorigenesis, the development of drug resistance, immune escape, and tumor microenvironment remodeling [30]. EVs primarily exert functional regulation through the delivery of DNA, mRNA, miRNA, long noncoding RNA, lipids, proteins, and carbohydrates [31]. Among these cargoes, miRNAs represent a class of small noncoding RNAs that bind to the 3 ^′^UTR of target mRNAs to induce translational repression or posttranscriptional degradation, thereby playing critical roles in biological processes such as cell migration, differentiation, apoptosis, and transformation [18]. Emerging evidence indicates that miRNAs serve as important diagnostic or prognostic biomarkers for malignant tumors and may represent potential therapeutic targets [32]. In NSCLC, miRNAs are extensively involved in dysregulated processes, although their precise functions in tumor proliferation, invasion, and metastasis remain unclear. For instance, certain miRNAs (e.g., miR‐21 and miR‐155) are highly expressed in NSCLC and promote tumor cell proliferation and metastasis, whereas others (e.g., miR‐34a and let‐7 family members) exhibit tumor‐suppressive effects, and their downregulation correlates with tumor progression and poor prognosis [33]. However, the regulatory mechanisms of the most aberrantly expressed miRNAs in NSCLC and their potential clinical applications require further investigation. Therefore, in‐depth exploration of miRNA functions and regulatory networks in NSCLC will not only advance our understanding of tumor pathogenesis but also identify novel diagnostic, prognostic, and therapeutic targets. By integrating high‐throughput sequencing technologies, bioinformatics analysis, and experimental validation, future research may identify clinically valuable miRNA biomarkers and therapeutic targets, ultimately improving the outcomes for patients with NSCLC.

The let‐7 family of miRNAs is recognized as a tumor suppressor family with critical regulatory roles in various cancers. Reportedly, let‐7 family members inhibit tumorigenesis and progression in multiple malignancies, including head and neck, breast, ovarian, and colorectal cancer [34]. Xi et al. demonstrated that hsa‐let‐7b‐5p suppresses glioma cell migration, invasion, and cell cycle progression [35]. Another study revealed that hsa‐let‐7e‐5p inhibited the progression of head and neck squamous cell carcinoma by targeting CCR7 expression [36]. In esophageal cancer, let‐7b‐5p negatively regulates KIAA1377 expression, thereby inhibiting tumor proliferation, metastasis, and invasion [37].

More importantly, multiple independent studies have confirmed that hsa‐let‐7b promotes tumor progression by inducing polarization of TAMs. Research has shown that PC3 prostate cancer cell line–derived EVs containing hsa‐let‐7b correlate with increased hsa‐let‐7b levels observed in TAMs [38]. Another study indicated that let‐7b‐5p may regulate M2 polarization through the SOCS1/STAT pathway and that let‐7b‐5p inhibitors reverse M2 differentiation while enhancing macrophage phagocytic activity, ultimately suppressing prostate cancer cell proliferation [39]. In acute myeloid leukemia (AML), knocking down let‐7b in TAMs repolarizes leukemia‐associated macrophages toward an M1‐like phenotype via activation of the Toll‐like receptor and NF‐*κ*B pathways, thereby inhibiting AML progression [40]. The antitumor properties of let‐7b‐5p have been increasingly exploited for antitumor drug development. A strategy combining TAM‐/TIDC‐targeted delivery with the dual biological functions of let‐7b (as both a TLR‐7 ligand and an IL‐10 inhibitor) may provide a novel approach for cancer immunotherapy [41].

Our current study revealed that miR‐let‐7b‐5p is significantly downregulated in lung cancer–derived exosomes and functions to inhibit the migration and invasion of NSCLC cells. At the same time, the miRlet‐7b‐5p mimic in EVs reversed the polarization of M2 macrophages, thereby inhibiting the migration, invasion, and EMT of NSCLC cells. Collectively, these data suggest that NSCLC cell–derived exosomal miR‐let‐7b‐5p plays a pivotal role in the tumor microenvironment, critically influencing tumor progression and metastasis.

AP1S1 is a critical regulator of intracellular vesicular transport and is primarily involved in protein trafficking and localization [42]. Recent studies have demonstrated abnormal AP1S1 expression in multiple malignancies, which is closely associated with tumorigenesis, tumor progression, and immune escape [43–45]. AP1S1 not only promotes tumor development through modulation of the activities of the cancer cells but also influences immune escape by regulating immune cells (e.g., macrophages, T cells, and dendritic cells) within the tumor microenvironment [44–48].

Studies have demonstrated that AP1S1 promotes tumor growth, invasion, and metastasis by regulating the function of TAMs. Additionally, AP1S1 modulates intracellular signaling pathways in macrophages (e.g., STAT3 and PI3K/AKT) [46], thereby promoting M2 polarization of macrophages and suppressing antitumor immune responses. Furthermore, AP1S1 influences tumor immune escape by regulating T‐cell functionality [49]. As an adaptor protein involved in vesicular trafficking [50, 51], AP1S1 may participate in regulating the intracellular transport and localization of E3 ubiquitin ligases (e.g., MDM2) or deubiquitinating enzymes that target p53, thereby indirectly influencing the ubiquitination modification and proteasomal degradation rate of p53. Studies have indicated that the AP family of proteins is involved in autophagosome formation [52, 53]. We speculate that the loss of AP1S1 may alter the turnover of p53 protein via the autophagy–lysosome pathway by affecting autophagic flux. In‐depth investigation of AP1S1′s mechanistic role in tumor immune escape will not only advance our understanding of the molecular pathways underlying immune evasion but will also identify novel therapeutic targets for immunotherapy. Future strategies targeting AP1S1 hold promise as significant breakthroughs in oncological treatments.

Collectively, exosomal miRNAs and miR‐let‐7b‐5p play pivotal roles in regulating macrophage polarization, modulating tumor immunity, and influencing malignant progression. AP1S1, a key regulator of intracellular vesicular transport, plays a critical role in cancer pathogenesis. Elucidating the regulatory mechanisms of these molecules within the tumor microenvironment will not only clarify the molecular mechanisms of tumorigenesis and progression but may also provide novel diagnostic and therapeutic strategies. Future studies are warranted to further explore the potential applications of these molecules in cancer immunotherapy and establish a theoretical foundation for personalized precision medicine.

## Ethics Statement

The authors have nothing to report.

## Consent

The authors have nothing to report.

## Disclosure

All authors read and approved the final manuscript.

## Conflicts of Interest

The authors declare no conflicts of interest.

## Author Contributions

L.L. and C.S. conceived and designed the study; L.L., J.A., Y.Z., and T.W. performed data analysis; Q.S., G.L., and Q.Z. contributed analysis tools; Z.K. and L.L. were the major contributors in writing the manuscript. L.L. and Z.K. contributed equally.

## Funding

This study was funded by the National Administration of Traditional Chinese Medicine High‐Level, ZYYZDXK‐2023125; the Taishan Scholars Program for Distinguished Experts of Shandong Province, tstp20221166; the Youth Project of Shandong Province Natural Science Foundation, ZR2023QH095; and the Weifang City Science and Technology Development Plan (University Category) Project, 2022GX008.

## Data Availability

The sequencing data supporting the findings of this study are available from the corresponding author, Changgang Sun (scgdoctor@126.com), upon request. Publicly available datasets were analyzed in this study. Data supporting the findings of this study are available in The Cancer Genome Atlas database at https://cancergenome.nih.gov/ and the Gene Expression Omnibus database at https://www.ncbi.nlm.nih.gov/geo/.
